# Beneficial Effects of GLP-1 Agonist in a Male With Compulsive Food-Related Behavior Associated With Autism

**DOI:** 10.3389/fpsyt.2019.00097

**Published:** 2019-03-01

**Authors:** Anna Järvinen, Merja K. Laine, Roope Tikkanen, Maija L. Castrén

**Affiliations:** ^1^Autism Foundation, Helsinki, Finland; ^2^Vantaa Health Center, Vantaa, Finland; ^3^Department of Psychiatry, University of Helsinki, Helsinki, Finland; ^4^Department of Physiology, Faculty of Medicine, University of Helsinki, Helsinki, Finland

**Keywords:** obsessive-compulsive disorder, autism, glucagon-like peptide-1 agonist, intellectual disability, weight, overeating

## Abstract

Individuals with autism spectrum disorder (ASD) frequently display intensely repetitive, restricted thoughts, and behaviors. These behaviors have similarities to compulsions and/or obsessions in obsessive compulsive disorder (OCD) and are primarily treated with behaviourally-based interventions and serotonin uptake inhibitors (SSRIs). Due to the lack of treatment responses in many cases, however, new treatments are being sought. Here we report beneficial effects of treatment with liraglutide, a glucagon-like peptide-1 (GLP-1) analog, on severe obsessive food craving, binge eating, weight gain, and behavioral problems in an adolescent male with infantile autism and moderate intellectual impairment. Liraglutide treatment reduced weight and unwanted behavior seemingly by preventing food-related repetitive thoughts and compulsions. Our report provides clinical evidence that GLP-1 signaling pathway may represent a novel target for treating food-related behavioral problems and aggressive behavior in ASD.

## Introduction

Restricted and repetitive thoughts and behaviors (RRBs) appearing inappropriate to the situation and odd in context represent some of the core diagnostic features in autism spectrum disorder (ASD) and associate with difficulties in interpreting everyday social signals and limited language and cognitive capabilities in ASD individuals (1). These are analogous to compulsions, which are driven by repetitive urge to perform the behavior and the tendency to repeat repetitive actions in a stereotyped or habitual manner (2). Voluntary control of compulsions is limited and the ability to delay or inhibit behaviors is diminished. Compulsions can significantly disturb the daily life and provoke far-reaching behavioral and functional problems. Obsessive compulsive disorder (OCD) is characterized by compulsions and/or obsessions that cause functional impairment (3). Rewarding effects followed by reduced anxiety caused by obsession link compulsion in OCD to behavioral addiction.

Differentiating RRBs of ASD from the obsessions and compulsions in OCD is challenging because of the heterogeneity of symptom manifestation and remarkable comorbidity characterizing both disorders. Particularly high-functioning persons with ASD have an elevated risk for OCD and co-occuring substance abuse (4). The high comorbidity and shared familial risks suggest that ASD and OCD-spectrum disorders have partially overlapping etiological mechanisms (5). Both ASD and OCD involve behavioral symptoms combined with cognitive manifestations (obsessions in OCD and insistence on sameness and preoccupations in ASD), which are presumed to be underpinned by anxiety, compromised executive functioning, and sensory phenomena. However, unlike in OCD, these behaviors often appear pleasurable and self-soothing in ASD, and the individuals may even behave aggressively if they are obstructed while performing the activities (5).

Cognitive behavioral therapy and serotonin uptake inhibitors (SRIs) are the first-line treatment for OCD (6). In the realm of pharmacological treatments for OCD symptoms in ASD, selective serotonin uptake inhibitors (SSRIs) have been reported to be beneficial (7). However, there is some evidence that individuals with ASD appear to be particularly sensitive to behavioral activation side effects of SSRIs (6). Antipsychotics provide therapeutic benefit for a subgroup of ASD individuals (8) but antipsychotic drugs also are disadvantaged by many side effects, including increased appetite, weight gain, and a subsequent increase in body mass index. Since treatment resistance OCD symptoms are common, several other treatment strategies have been tested. Abnormalities identified in glutamatergic neurotransmission in OCD have led to clinical trials with “glutamate-modulating” drugs (9). In this line of studies anti-convulsive drugs (topiramate and lamotrigine), D-cycloserine, memantine, minocycline, modafinil, N-acetylcysteine, and riluzole have been associated with beneficial therapeutic effects in OCD but their clinical utility has not been proven in relatively few studies with partially inconsistent results.

Food-related obsessions and compulsive overeating represent some of the landmark characteristics of Prader Willi syndrome (PWS). Preliminary evidence indicates that glucagon-like peptide-1 (GLP-1) receptor agonists could be used to treat defects of satiety responses in this genetically based intellectual disability syndrome (10–12). Similarly to PWS, both children and adults with ASD suffer more frequently from obesity than the general population. Here we report beneficial effects of liraglutide, a human GLP-1 analog, on food-related obsessions and compulsive eating in a case with a neurodevelopmental disorder characterized by autistic features. No adverse side effects of liraglutide were observed in our study/patient case.

## Case Report

A 20-year-old Finnish male patient is the second-born child of healthy, non-consanguineous parents with an unremarkable family history. His close relatives had no manifestations of thyroid or heritable endocrine diseases. The perinatal period was uneventful, however, deficits in eye contact behavior were noted from early infancy onwards. By the preschool age, he demonstrated behavioral difficulties resembling those associated with autism, including perseveration and impairments in social interactive behavior including avoidance of strangers. Abnormal responses to auditory, olfactory, and oral sensory stimuli were noted. Motor and phonic tics as well as obsessions appeared in adolescence being periodically severe. At the age of 6;9 years, the patient received the diagnoses of pervasive developmental disorder-not otherwise specified and mild intellectual impairment, with these later, at the age of 11;2 years, having been modified to infantile autism and moderate intellectual impairment. Behavioral problems included aggressive behavior, which resulted in treatment with risperidone being initiated at the age of 12 years. Initially the antipsychotic slightly appeared to reduce behavioral difficulties while at the same time resulting in rapid weight gain and nightmares. Within 6 months, risperidone was substituted with aripiprazole. Aripiprazole caused initially fatigue, muscular spasms of jaw, and slurring of speech at the dose of 5 mg/day. After a break for several months, aripiprazole treatment was continued and the dose was slowly increased to 7.5 mg/day. Agitation and disturbing daily RRBs such as switching on and off a water tap, checking, and jumping led to the combining of citalopram to the medication at the age of 14;10 years. Nevertheless, the gradually increased dose of citalopram to 20 mg/day did not improve the situation and especially food-related obsessions and constant weight gain appeared problematic. Craving of food, particularly sweet drinks, led the patient to e.g., steal food. A temporary increase in alanine transferase (ALT; 77 U/L, reference range <40 U/L) together with a slight decrease in thyroxin (T4) levels (11 pmol/L, reference range 12–20 pmol/L) were observed. In the laboratory tests prior to commencing the liraglutide treatment, ALT was diminished (56 U/L), γ-glutamyltransferase (γ-GT) normal (<50 U/L), serum TSH 1.3 mU/L (reference range 0.2–4.2 mU/L) with the values for lipid metabolism, blood count, creatinine, and fasting glucose being within the normal range. In metabolic screening, urine amino acids, oligosaccharides, and glycosaminoglycans were within the normal range, similarly EEG was normal. Further, karyotyping and fragile X studies resulted in normal findings. Ophthalmological examination revealed hyperopia (+5.0/+5.0) that was treated with glasses. Hearing was normal in the otoacoustic emissions test.

Within the cognitive domain, the patient's cognitive functioning was commensurate with the level of moderate intellectual impairment (full scale intelligence quotient 43) at the age of 19;3 years. His verbal comprehension, perceptual reasoning, and processing speed indices were at the very poor level (50, 50, and 64, respectively), with the working memory index being slightly better (71). In terms of memory, rote learning and digit span were within the normal range with all other functions being notably compromised. His level of autistic symptoms as assessed across lifespan were significantly elevated (Social Communication Questionnaire life-time version score 24). Similarly, his level of social functioning was moderately impaired (Social Responsiveness Scale T-score 69, with most pronounced deficits seen in social cognition and autistic mannerisms). In the Strengths and Difficulties Questionnaire as responded by parents, hyperactivity and friendship scales resulted in aberrant scores. There were also significant OCD symptoms as assessed by the OCI-R (29 points). In terms of adaptive functioning, results from the Vineland-II Adaptive Behavior Scales at the age of 19;3 years indicated a low level of functioning overall, with the following mental age equivalents for subdomains: receptive communication 6;6, expressive communication 12;3, written communication 15;3, personal daily living skills 10;6, domestic daily living skills 9;6, community daily living skills 13;00, interpersonal relationships 3;10, play and leisure time 4;7, coping skills 7;1. In addition, both internalizing and externalizing maladaptive behaviors were at a clinically significant level. In childhood, the patient's rehabilitation has included both speech therapy and occupational therapy.

**Figure 1:**
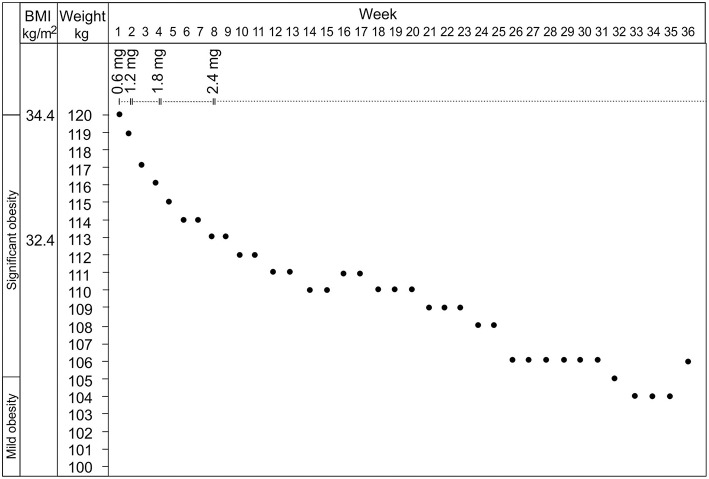
The effects of liraglutide at the dosage from 0.6 to 2.4 mg on the body weight and the body mass index of the patient.

Treatment with liraglutide was initiated with a dose of 0.6 mg/day and being gradually increased to 2.4 mg/day during the following 8 weeks. Immediate positive response was observed in the patient's food-related behavior manifesting as drastically subsided obsessive food-related thoughts, craving for food, and compulsive eating. After first week of treatment, a clear reduction in patient's body weight was seen (Figure 1). Also obsessions, compulsions and behavioral problems not related to food, including aggressive behavior, decreased in a significant way at home. The treatment was continued 36-weeks with the dose 2.4 mg/day. At the time-point 8 weeks, the weight was already reduced by 6%. From week 25 to the end of the follow-up the weight reduction settled at 12–13%. In the laboratory control at 8 weeks, the standardized oral glucose tolerance test was normal (glucose 5.3 and 4.6 mmol/L before and 120-min after the glucose administration, respectively). In later control fasting glucose and insulin levels were normal. No adverse side effects of liraglutide were observed in our patient case.

## Discussion

Relevance of the ability of GPL-1 to promote satiety and reduce food intake in connection with compulsive eating behavior has recently attracted attention [see review of GLP-1 (13)]. GLP-1 belongs to the group of gut-derived peptide hormones referred to as incretins, which are released in response to oral intake of nutrients. Native GLP-1 is rapidly degraded in circulation, and therefore, long-acting GLP-1 receptor agonists have been developed, allowing once daily to once weekly administration as subcutaneous injection. GLP-1 agonists have been shown to be effective in the pharmacotherapy of obesity and type 2 diabetes (14). In the current study, we found that liraglutide remedied food-related obsessions and related disruptive behavior in our patient case with ASD. The patient's obsessions to food initially emerged following the treatment with atypical antipsychotics. Antipsychotic drugs affect several neurotransmitter systems implicated in both motivational and regulatory aspects of food intake, insulin release, and glucose metabolism. It has also been postulated that centrally-mediated vagal efferent activity, regulated via hypothalamus through repetitive and increased food ingestion as well as via increased circulating free fatty acids contribute to the effects of atypical antipsychotics (15). The rapid weight gain caused by antipsychotics did not associate with alterations of serum lipids in our patient case, albeit marginal increases in alanine amino transferase as well as reduced serum thyroxine concentration during risperidone treatment suggested some metabolic changes. However, the values had normalized before the treatment with GLP-1 analog, which was used in combination with aripiprazole and citalopram. There was no evidence that liraglutide had an interaction with the patient's other medication.

According to the parental reports, the patient case also demonstrated food-independent RRBs which fluctuated in severity but appeared daily and very disturbing prior to the onset of the liraglutide treatment. Notably, liraglutide ameliorated these behaviors as well as antisocial and challenging behavior that was initially the focus of the treatment with antipsychotics, suggesting beneficial effects of liraglutide treatment also on impulse control. The substantial decrease in weight during liraglutide treatment likely has several underlying causal mechanisms paralleling results of addiction research describing compulsive substance use and reward seeking behavior (16). GLP-1 receptor activation decreases anticipatory food reward and increases consummatory food reward, which may reduce cravings for food and prevent overeating, respectively (17). Direct cognitive-behavioral effects of GLP-1 analog by altering the reward circuits of the brain have been shown in several addictions with inherent compulsive substance usage (16). There is evidence that GLP-1 signaling in hippocampus enhances learning and memory, as well as plays a role in protecting brain from seizures and neuronal damage via safeguarding against amyloid-beta peptide induced cognitive decline (18). It is thus possible that in our patient case the administration of liraglutide also influenced some cognitive processes and altered reward circuits of the brain in addition to the physiological satiety-promoting effects. However, the direct effects of liraglutide on cognition were not measured in our study.

Both ASD and OCD are characterized by poor behavioral inhibition and executive functioning, which are associated with dysfunction in cortico-striatal-thalamic circuits (19). GLP-1 is expressed in a subpopulation of hindbrain neurons, which send axons to many forebrain regions, including the paraventricular nucleus (PVN) of the hypothalamus. Effects of GLP-1 on feeding behavior are mediated via GLP-1 receptors (GLP-1R) in PVN neurons (20). However, GLP-1R ablation in PVN does not affect glucose metabolism and the vagus nerve is implicated in the effects of peripherally administered GLP-1 on food intake and glycaemia (21). GLP-1Rs are expressed in vagal afferent neurons which terminate in the lamina propria of the intestinal mucosa and vagal afferents may transfer the gut GLP-1–derived signals to the brain and mediate satiating and glucoregulatory responses. Altogether, neuronal mechanisms that contribute to the beneficial effects of GLP-1 analog on the behavioral challenges in ASD remain to be further studied.

## Concluding Remarks

The present study points to GLP-1 signaling as a potential target for treating food-related behavioral problems in intellectual disability syndromes with ASD. As our findings are limited to a single patient case further studies are warranted to further explore the therapeutic utility of GLP-1 analog in neuropsychiatric symptoms in a sizeable population and randomized controlled trials.

## Ethics Statement

The study was performed in accordance with the Declaration of Helsinki and a written consent was obtained from both the patient and his caretaker authorizing the publication of the clinical case report. The anonymity of the patient case has been preserved.

## Author Contributions

MC designed the study and together with AJ managed the literature searches and analyses. MC wrote the first draft of the manuscript. All authors contributed to and have approved the final manuscript.

### Conflict of Interest Statement

The authors declare that the research was conducted in the absence of any commercial or financial relationships that could be construed as a potential conflict of interest.
